# Changes in effective connectivity during the visual-motor integration tasks: a preliminary f-NIRS study

**DOI:** 10.1186/s12993-024-00232-3

**Published:** 2024-03-11

**Authors:** Wenchen Wang, Haimei Li, Yufeng Wang, Lu Liu, Qiujin Qian

**Affiliations:** 1 Peking University Sixth Hospital, Institute of Mental Health, Beijing, 100191 China; 2 NHC Key Laboratory of Mental Health (Peking University), National Clinical Research Center for Mental Disorders (Peking University Sixth Hospital), Beijing, 100191 China

**Keywords:** Visual-motor integration, f-NIRS, Effective connectivity, Executive function, Attention

## Abstract

**Background:**

Visual-motor integration (VMI) is an essential skill in daily life. The present study aimed to use functional near-infrared spectroscopy (fNIRS) technology to explore the effective connectivity (EC) changes among brain regions during VMI activities of varying difficulty levels.

**Methods:**

A total of 17 healthy participants were recruited for the study. Continuous Performance Test (CPT), Behavior Rating Inventory of Executive Function-Adult Version (BRIEF-A), and Beery VMI test were used to evaluate attention performance, executive function, and VMI performance. Granger causality analysis was performed for the VMI task data to obtain the EC matrix for all participants. One-way ANOVA analysis was used to identify VMI load-dependent EC values among different task difficulty levels from brain network and channel perspectives, and partial correlation analysis was used to explore the relationship between VMI load-dependent EC values and behavioral performance.

**Results:**

We found that the EC values of dorsal attention network (DAN) → default mode network (DMN), DAN → ventral attention network (VAN), DAN → frontoparietal network (FPN), and DAN → somatomotor network (SMN) in the complex condition were higher than those in the simple and moderate conditions. Further channel analyses indicated that the EC values of the right superior parietal lobule (SPL) → right superior frontal gyrus (SFG), right middle occipital gyrus (MOG) → left SFG, and right MOG → right postcentral gyrus (PCG) in the complex condition were higher than those in the simple and moderate conditions. Subsequent partial correlation analysis revealed that the EC values from DAN to DMN, VAN, and SMN were positively correlated with executive function and VMI performance. Furthermore, the EC values of right MOG → left SFG and right MOG → right PCG were positively correlated with attention performance.

**Conclusions:**

The DAN is actively involved during the VMI task and thus may play a critical role in VMI processes, in which two key brain regions (right SPL, right MOG) may contribute to the EC changes in response to increasing VMI load. Meanwhile, bilateral SFG and right PCG may also be closely related to the VMI performance.

**Supplementary Information:**

The online version contains supplementary material available at 10.1186/s12993-024-00232-3.

## Background

Over the past few decades, there has been increasing interest in studying how the brain links sensory perception to movement [1], such as the integration of visual perception and motor control [2]. Visual-motor integration (VMI) refers to the mutual coordination ability to coordinate visual perception and motor output during the purposeful activities of individuals [3]. VMI skills involve hand–eye coordination, visual perception skills, and fine motor coordination. Visually guided motor movements are essential for many aspects of daily life, work, and learning (e.g., throwing a ball, handwriting, copying a shape, or drawing a figure) [4]. The VMI difficulties were characterized by impaired handwriting and sloppy figure copying [5].

The VMI is an important internal factor that affects individual fine motor skills and reflects the development and maturity of the brain to a certain degree. In the process of visual information transmission, there are two main transmission pathways: the ventral pathway and the dorsal pathway [6]. The dorsal pathway transmits visual information from the primary visual cortex (V1) to the parietal and frontal lobes, which use visual information to understand motion and spatial layout, translating this information into movement control [7]. According to previous studies, the dorsal pathway is closely associated with VMI function [8, 9]. The VMI deficit in developmental disorders such as attention-deficit/hyperactivity disorder (ADHD) and autism spectrum disorder (ASD) may be related to the atypical development of the dorsal pathway, called ‘Dorsal Stream Vulnerability’ [10, 11].

Based on resting-state and task-state (finger tapping task) fMRI, Bueichekú et al. characterized the functional network of the VMI system in healthy individuals. It was found that the medial occipital region, intraparietal sulcus, motor cortex, and parietal insula may be related to the integration of the visual and motor systems [12]. Similarly, using the stepwise functional connectivity analysis of resting-state fMRI study, Sepulcre found that the superior parietal lobule (SPL), the parietal insula, the anterior insula/ventral premotor area, may be associated with the ability to visuomotor integrate [13]. A fNIRS-based study showed that adaptive visuomotor task with high ecological validity can enhance effective connectivity (EC) between the prefrontal and sensorimotor areas [14].

Completing VMI movements involves not only coordination between hand movement control and the eyes, but also complex cognitive processes such as planning, task flexibility, goal orientation, response inhibition, and maintaining attention throughout the task [15, 16]. Hence, the executive function (EF) may be closely related to VMI [17]. Notably, the association between EF and VMI performance still requires further clarification. It was also found that the brain regions through which visual information is transmitted in the dorsal pathway overlap with attentional control regions [7]. Barton et al. found a significant increase in functional connectivity between SPL/anterior intraparietal sulcus and primary motor cortex, during letter writing compared to a simple dot-writing task, the results suggested that the increased functional connectivity may related to the difficulty of the writing task and the increase in motor attention demands [18]. Therefore, attention control may have an important role in the VMI process.

The Beery-Buktenica Developmental Test of Visual-Motor Integration (Beery VMI) is one of the most used standardized measures of VMI function [19]. However, traditional VMI tests have mainly focused on assessing the results of paper-and-pencil tests; computer-based and digital technologies have facilitated the development of evaluation innovations. Current computerized assessment methods for these VMI are limited to observations and descriptions of behavioral performance. Based on the Beery VMI test, Wee et al. proposed a 4D dynamic analysis system that implements VMI testing in a 3D virtual space and obtains time-series data of hand joints and trajectories [20]. Nicholas et al. used eye-tracking technology to observe the eye movements of children during the Beery VMI test, providing a new method for assessing visual-motor integration in real time [21]. However, currently, there is no research that synchronously observed changes in functional brain activity during the Beery VMI task. Understanding the neural mechanisms underlying VMI can help provide interventions for individuals with VMI difficulties. In addition, previous studies suggested that EC analysis can more accurately determine causal relationships between brain regions, and thus, measures how one brain region influences another in a specific direction, whereas unidirectional functional connectivity analysis only provides information on the correlation between brain regions [22].

## Purpose of this study

Given the above, some significant questions still remain. During the Beery VMI test, do EC values between different brain regions change with increasing task difficulty? Are these changes associated with behavioral performance? Our present study attempted to use the functional near-infrared spectroscopy (fNIRS) technology to evaluate VMI load-dependent EC values of the brain regions in healthy adults during the VMI tasks, and preliminarily explore the relationship of these observed EC values with VMI performance attention performance and EFs.

## Methods

### Participants

We recruited 23 healthy volunteers (mean age 24.74 ± 3.00; 10 males) for our study. The inclusion criteria for all participants were: (a) above 18 years; (b) full-scale intelligence quotient (FSIQ) score of ≥ 90; and (c) right-handed. The Structured Clinical Interview for Diagnostic and Statistical Manual of Mental Disorders IV Axis I disorders (SCID) [23], the Conner’s Adult ADHD Diagnostic Interview [24], the Hamilton Anxiety Scale, and the Hamilton Depression Rating Scale [25] were used to determine the presence or absence of psychiatric disorders. The detailed demographic and clinical characteristics can be found in Additional file 1: Table S1. The protocol was approved by the Ethics Committee of Peking University Sixth Hospital/Institute of Mental Health. We obtained informed consent from all participants.

### Assessment

#### VMI performance

The Beery VMI (Fourth edition) comprises 24 geometric designs that increase in difficulty. The participants were asked to copy geometric designs with paper and pencil. Scores are based on the accuracy with which the designs were copied. The higher scores indicate better VMI performance [26, 27].

#### Attention performance

The Continuous Performance Test-Identical Pairs (CPT-IP) is used to evaluate sustained attention, selective attention, and vigilance. During the test, participants are presented with a series of numbers on a screen for a brief period, composed of three conditions (2-digit, 3-digit, and 4-digit). They must monitor the numbers and respond by pressing a key when two consecutive stimuli are identical. Meanwhile, the computer automatically records the response data of participants. Performance is measured using the detection index (d′ statistic), with higher d′ values indicating better performance for attention and vigilance [28].

#### Executive function (EF)

The Behavior Rating Inventory of Executive Function-Adult Version (BRIEF-A) was adopted for the questionnaire-based scale. It is a self-rating scale (75 items) that assesses the ecological EF of adults. The scale generates two broad indices (nine factors): the Metacognition Index (MI, including Working memory, Initiate, Plan/Organize, Organization of Materials, and Task Monitor subscales) and the Behavioral regulation index (BRI, including Inhibition, Shift, Emotional Control and Self-Monitor subscales) [29]. The Chinese version of the BRIEF-A had adequate criterion validity (r = 0.39–0.78) and test–retest reliability (r = 0.61–0.76) [30]. Higher scores indicate more severe EF impairment.

### Tasks and procedures

#### Experimental setup

**Fig. 1:**
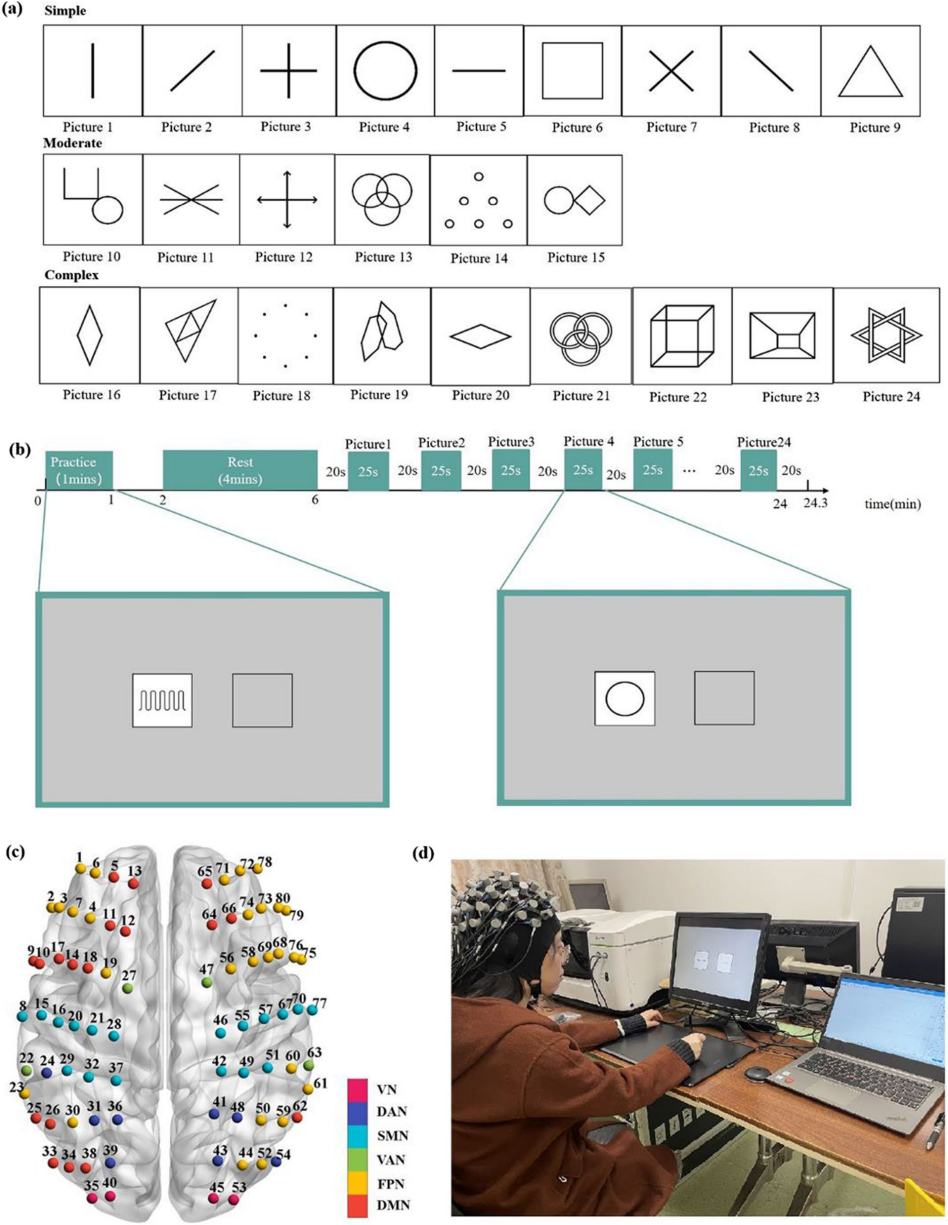
Experimental setup. **a** The task pictures of the VMI test. **b** Overview of experimental design set up. **c** Positions of f-NIRS channels. **d** Photo obtained from a participant during the VMI task. *DMN* default mode network, *DAN* dorsal attention network, *VAN* ventral attention network, *FPN* frontoparietal network, *SMN* somatomotor network, *VN* visual network

Visual-Motor Integration (VMI) Tasks: all pictures of the VMI task were adopted from the Beery VMI test (paper and pencil test), and the task pictures (Picture 1–Picture 24) were presented in Fig. 1a. Based on previous research [21], our study divided the 24 pictures into three difficulty levels: simple, moderate, and complex. The pictures were presented in order of difficulty from simple to moderate and then to complex. The traces of drawing pictures were recorded on a digitizing tablet connected to a desktop computer (a resolution of 1280 × 1024 pixels and a 60 Hz refresh rate). They were saved as image files (with.png file format). The square size on the computer screen was the same as the paper version of the Beery VMI test (6 cm × 6 cm). The active area of digitizing tablet is 21.6 cm × 21.6 cm, and the presentation of experimental geometric pictures and the recording of behavioral data were all performed by MATLAB (R2013b) scripts. We divided this task into two parts: practice session and testing session. In the practice session, all participants performed two practice pictures (different from the testing pictures, see Additional file 1: Fig. S1) to familiarize the testing session of the VMI task. During the testing session, 24 different pictures appeared sequentially on the white square (left side of the computer screen); participants were asked to draw the geometric pictures repeatedly into a gray square (right side of the screen) on a digitizing tablet (Wacom Intuos pro-PTH 860/K1-F, Japan) using an electronic pen. Once the electronic pen touched the digitizing tablet, the gray square disappeared and was replaced with a white square with a black border around it. A block design was used to ask the participants to perform the geometric pictures stimulation for 25 s followed by 20 s rest. Hence, the total length of the task was approximately 24 min. Detailed information of the experimental set up is presented in Fig. 1b.

Participants were reminded to draw the pictures as quickly and accurately as possible in the testing session. An independent investigator evaluated the accuracy of writing performance according to the standard scoring criteria of the Beery VMI test. All figures were presented for a duration of 25 s and were required to be completed at least once within this timeframe. For complex figures, if they could not be completed within the timeframe (25 s), the corresponding data were excluded from the data analysis. In addition, previous research [20] found that using the 4D system, healthy adults required an average of 50.72 ± 22.22 s and 51.16 ± 18.15 s to complete Picture21 and Picture24, respectively. Thus, participants may not have been able to complete a single drawing within the 25 s. Consequently, data corresponding to these two figures were not used for the subsequent data processing. Observation time, total drawing number, and drawing speed was used to record the experimental procedure. Observation time (information-gathering phase) was defined as the time duration from the onset of the trial to the beginning of drawing [21]. The total drawing number was defined as the number of pictures drawn by the subject in each trial. The drawing speed was defined as the total travel distance on the tablet divided by the total time in each trial [31].

#### fNIRS data acquisition

During the VMI task, we used the multichannel near-infrared optical imaging system (NirScan-6000A, Danyang Huichuang Medical Equipment Co., Ltd., China) to acquire fNIRS data simultaneously. The system has 52 optical poles, including 24 light sources (two wavelengths: 670 and 830 nm) and 28 detectors, with a sampling rate of 17 Hz. The source-detector distance was fixed at 3 cm, and a total of 80 channels were generated, covering the parietal, frontal, temporal, and occipital lobe regions of the brain.

#### MRI coregistration

To confirm the positions of each measurement channel, we randomly choose a participant for structural MRI scanning. We labeled all the source-detector positions of the fNIRS cap using the vitamin E capsules, and the participant was scanned on the 3T MRI Scanner (Discovery 750) while wearing the fNIRS cap [32]. After obtaining the spatial coordinates information of each channel, we divided the whole brain into six functional networks, including the frontoparietal network (FPN), dorsal attention network (DAN), ventral attention network (VAN), somatomotor network (SMN), visual network (VN), and default network (DMN), according to Yeo et al.’s network template [33]. The process of obtaining Montreal Neurological Institute (MNI) coordinates, and the distribution of all NIRS channels in the six brain networks are presented in Fig. 1c, d and Additional file 1. A similar method of positioning was also used in previous studies [34].

#### fNIRS data preprocessing

The data preprocessing was performed by the MATLAB-based toolbox–Homer2 [35]. The procedures were as follows: (a) the raw light intensity was converted to optical density (OD) signal; (b) the motion artifacts were detected by automatic artifact inspection (tMotion = 0.6, tMask = 1, STDEVthresh = 20, AMPthresh = 2); (c) Motion artifacts correction (hmrMotionCorrectSpline: p = 0.99); (d) Band-pass filter (hmrBandpassFilt: 0.01–0.1Hz); (e) Convert OD signal to oxygenated hemoglobin (HbO) and deoxygenated hemoglobin (Hb) concentrations using the Beer–Lambert law. However, we only considered HbO concentration for the statistical analyses because of its better signal-to-noise ratio compared to Hb concentration [36]; and (f) Block average: we set [− 2, 35] as the time window after the onset of the testing session to calculate the mean concentration of HbO [37].

### Data analysis

#### Behavioral data analysis

The demographic characteristics of participants were summarized using descriptive analyses, such as means, standard deviations, and frequencies. Behavioral data (observation time, total drawing number, and drawing speed) were analyzed by one-way ANOVA and corrected for the post-host test with the Bonferroni correction. All behavioral data analyses were performed by IBM SPSS 25.0. The significance level was set at *P* < 0.05.

#### fNIRS data analysis

EC: was measured using G-causality analysis, which is considered to apply to time-series data and can reflect the direction of information flow between different brain regions during task performance [38]. In our study, G-causality analysis was processed by the Hermes Toolbox [39], which was implemented in MATLAB. A vector auto-regressive model was established to calculate EC values, and the best model order ‘p’ was identified using the Akaike [40] and the Bayesian Information Criterion [41, 42]. For two continuous time series x(t) and y(t), if the accuracy of the model using the past information of y(t) and x(t) is higher than the model only using x(t), it can be considered that y(t) is assumed to cause x (t) (there is an information flow from y(t) → x(t)) [43].

Through the G-causality analysis, we obtained the EC values for each channel under three different conditions (from the perspective of brain regions). Based on the network template, the network EC values was obtained by average EC values across all channels within each network (e.g., DAN including 8 channels, the network EC values of DAN is the average of the EC values of the 8 channels), resulting in three 6*6 EC value matrices. The subsequent statistical analyses are based on the EC values for each network or brain region.

In our present study, we have conducted ANCOVA analyses at the network level and channel level subsequently. Firstly, we intended to find the difference EC values among the three conditions at the network level, with gender, age, and total IQ as covariates (ANCOVA analysis); and then, based on the network difference, we further performed ANCOVA analysis to explore the difference at the channel level which may help us to elucidate the specifically involved brain regions. Then, partial correlation analysis between EC values and behavioral data were performed from the network perspective and the channel perspective respectively. False discovery rate (FDR) correction was used to minimize the multiple comparison problems. Partial correlation analysis was performed to investigate the correlation of the EF, VMI performance, and EC values with sex, age, and FSIQ as covariates. The flow chart for the GC analyses is presented in Additional file 1: Fig. S2.

All statistical analyses for fNIRS data were conducted by MATLAB script and SPSS 25.0, and all 3-D brain figures were visualized using the BrainNet Viewer Toolbox [44].

## Results

### Behaviors data

In order to guarantee the validity of the results, only those subjects who drew the pictures accurately at least once were selected for analysis. Data from 17 subjects were finally included for the analyses. However, in all these subjects, the drawing of Picture 21 and Picture 24 could not be completed within the time limit. Consequently, we removed these two pictures from the analysis, and the remaining 22 pictures were included in the subsequent behavioral and fNIRS data analysis. The results of the inaccurate drawing parts (Picture 21 and Picture 24) were presented in Additional file 1: Fig. S3.

Using the One-way ANOVA, we found a significant difference in the total drawing number (*P* = 5.799E−9) and drawing speed (*P* = 0.016) among the three conditions. No significant difference was found in the observation time among the three conditions (*P* = 0.290).

Post hoc tests revealed that the total drawing number of simple conditions was significantly higher than moderate conditions (*P* = 2.662E−7) and complex conditions (*P* = 3.907E−8), respectively. Concerning the drawing speed, after the post hoc test, the drawing speed of simple pictures was significantly higher than that of complex conditions (*P* = 3.907E−8) (Table 1).

**Table 1:** The mean scores of the behavior performance in three conditions

Mean scores	Simple	Moderate	Complex	t	*P*	Post hoc (*Bonferroni*)
Observation time (s)	1.834 ± 0.397	1.758 ± 0.413	1.991 ± .490	1.113	0.290	–
Total drawing number	10.569 ± 4.515	4.530 ± 1.511	4.009 ± 0.895	38.962	5.799E−9	1 > 2, 3
Drawing speed (cm/s)	5.225 ± 2.983	4.255 ± 1.81	3.056 ± 1.047	6.648	0.016	1 > 2

### G-causality analysis

Through data preprocessing analysis, we obtained the blood oxygen concentrations of HbO and HbR and performed G-causality analysis only on the basis of HbO concentration. The group level of time course of the fNIRS response (both Hbo and HbR) is presented in Additional file 1: Fig. S4.

### The EC values between the three conditions in brain networks

**Fig. 2:**
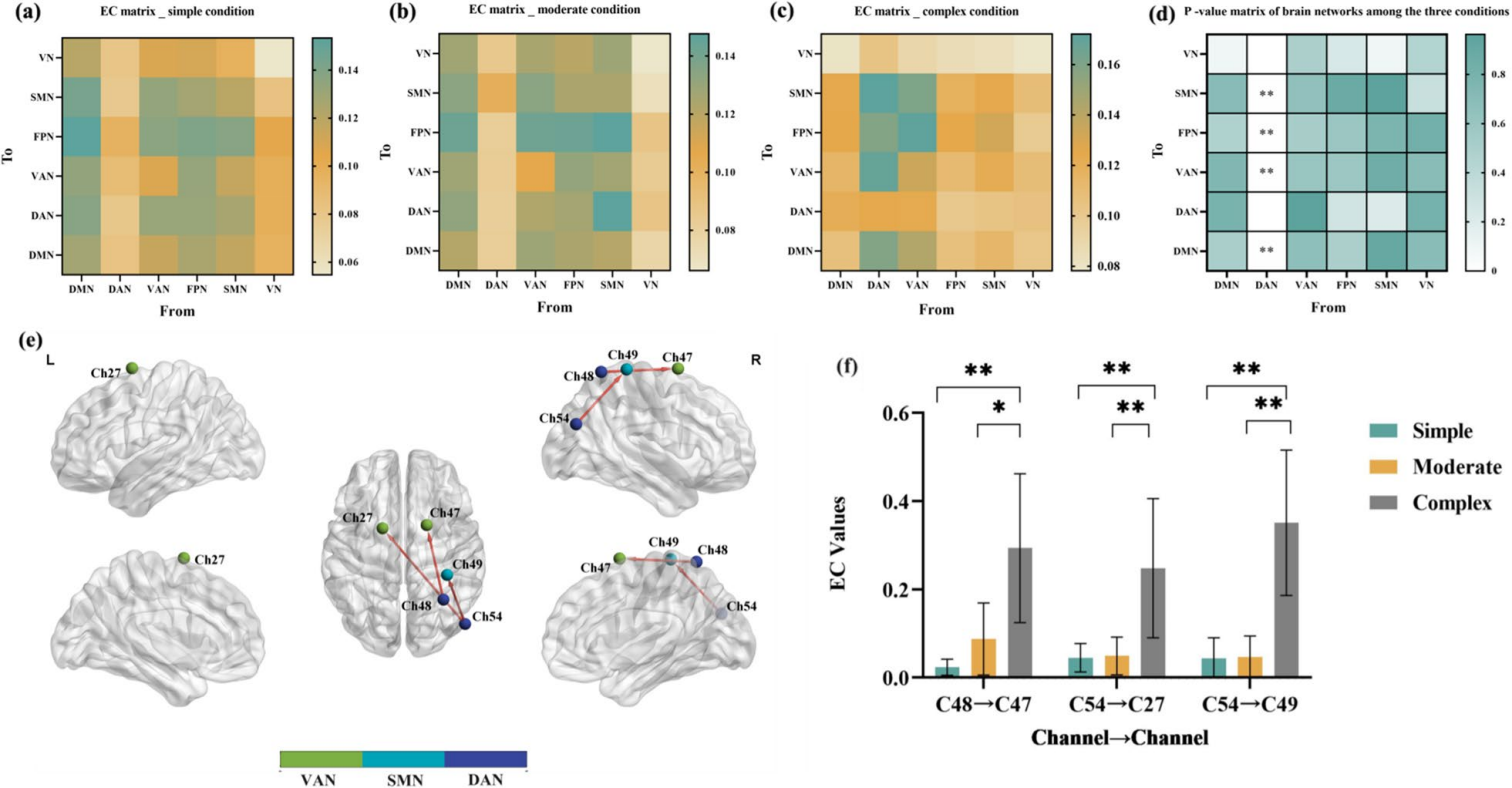
Effective connectivity changes of brain networks and channels in three conditions. **a** EC matrix of all brain networks in simple condition; **b** EC matrix of all brain networks in moderate condition; **c** EC matrix of all brain networks in complex condition; **d** P-value matrix of brain networks among the three conditions (the white square in the P-value matrix represents no statistical differences in results, while the blue one represents significant differences); **e**, **f** significantly different EC values of channels among the three conditions. *Significant differences survived FDR correction for multiple testing (*P < 0.05, **P < 0.01). *DMN* default mode network, *DAN* dorsal attention network, *VAN* ventral attention network, *FPN* frontoparietal network, *SMN* somatomotor network, *VN* visual network

Using the G-causality analysis, we obtained the averaged EC values matrix of six brain networks (Fig. 2a–c). To examine whether there were significant differences in the network EC values among the three conditions, ANCOVA was performed to compare the EC values of each network metrics among the three conditions, with controlling for sex, age, and FSIQ. We found that there were significant differences in the averaged EC values of the DAN → DMN (*P*_FDR_ = 0.002), the DAN → VAN (*P*_FDR_ = 0.003), the DAN → FPN (*P*_FDR_ = 0.004), the DAN → SMN (*P*_FDR_ = 0.003) (Fig. 2d, the blue squares represent the significant differences). The detailed information is presented in Additional file 1: Table S3.

Based on the results of ANCOVA, the post hoc test found that the EC values of the DAN → DMN, DAN → VAN, DAN → FPN, DAN → SMN in the complex condition were higher than that in the simple and moderate conditions, which represented that there was an increase in the EC values from DAN to DMN, from DAN to VAN, from DAN to FPN, and from DAN to SMN with the increase of the task load. However, the post hoc test found no significantly different EC values between simple and moderate conditions (see Additional file 1: Fig. S5).

### The EC values between the three conditions in channels

In the results of network analysis, we found that there were five significant different network EC values (the DAN → DMN, the DAN → VAN, the DAN → FPN, the DAN → SMN) among three conditions; And then we further obtained the EC values matrix for each channel of the five significant different network values matrix (see Additional file 1: Fig. S6).

ANCOVA was conducted on the specific channel of the networks EC values with significant differences, with controlling for sex, age, and FSIQ. The results showed that after the FDR correction, only the connectivity of the C48 → C47 (*P*_FDR_ = 0.001), the C54 → C27 (*P*_FDR_ = 0.003), and the C54 → C49 (*P*_*FDR*_ = 2.499E−05) were significantly different between the three conditions (Fig. 2e).

Based on the results of FDR correction, the post hoc test found that the EC values of the C48 → C47, C54 → C27, and C54 → C49 in the complex condition were higher than in the simple condition and moderate condition. No significantly different EC values of channels were found between simple and moderate conditions (Fig. 2f). The detailed information is presented in Additional file 1: Table S4.

### The correlation of EF and attention performance with EC values

Based on the post hoc test results, we found no significant differences in the EC values of the different networks at the simple and moderate conditions. Hence, we merged the simple and moderate conditions, and used the mean values of the simple and moderate conditions for partial correlation analysis.

In the BRIEF scales, the EC values from DAN to other networks were positively correlated with Emotional control, Task Monitor, and Organization of Materials factors, respectively. In detail, the EC values of DAN → VAN were positively correlated with the Emotional control (r = 0.598, *P* = 0.024) and MI (r = 0.556, *P* = 0.039) in the simple + moderate conditions. Meanwhile, in complex conditions, the EC values between DAN → DMN (r = 0.544, *P* = 0.044) and DAN → SMN (r = 0.538, *P* = 0.047) were positively correlated with the Organization of Materials (Table 2). From the perspective of channels, no significant correlation was found between the subscales of BRIEF and EC value changes.

**Table 2:** The correlation between BRIEF, VMI, CPT and EC values of networks in different conditions

Conditions	Networks	BRIEF r (*P*)											VMI scores	CPT r (*P*)		
		WM	Initiate	Plan/organize	OM	TM	Inhibit	Shift	EC	Self-monitor	MI	BRI		2-digit	3-digit	4-digit
Simple + moderate	DAN → DMN	0.274 (0.343)	− 0.152 (0.605)	0.117 (0.689)	0.021 (0.944)	− 0.060 (0.838)	0.209 (0.474)	0.227 (0.436)	0.213 (0.465)	0.209 (0.474)	0.240 (0.409)	0.040 (0.892)	− 0.048 (0.895)	0.227 (0.436)	0.036 (0.902)	− 0.195 (0.505)
	DAN → VAN	0.410 (0.146)	0.322 (0.262)	0.481 (0.082)	− 0.078 (0.791)	− 0.149 (0.612)	0.403 (0.153)	0.484 (0.079)	**0.598** **(0.024)**	0.433 (0.122)	**0.556** **(0.039)**	0.270 (0.351)	− 0.603 (0.065)	0.021 (0.942)	− 0.106 (0.718)	− 0.115 (0.697)
	DAN → FPN	0.191 (0.513)	− 0.244 (0.400)	− 0.005 (0.987)	− 0.119 (0.686)	− 0.276 (0.340)	0.080 (0.785)	0.169 (0.563)	0.090 (0.760)	0.024 (0.934)	0.109 (0.710)	− 0.121 (0.682)	− 0.139 (0.701)	0.177 (0.546)	0.128 (0.664)	− 0.186 (0.524)
	DAN → SMN	0.143 (0.626)	− 0.373 (0.189)	− 0.177 (0.544)	− 0.270 (0.350)	− 0.275 (0.340)	− 0.072 (0.807)	0.100 (0.735)	− 0.063 (0.831)	− 0.115 (0.695)	− 0.031 (0.916)	− 0.259 (0.372)	− 0.145 (0.689)	0.267 (0.357)	0.201 (0.490)	− 0.215 (0.460)
Complex	DAN → DMN	0.411 (0.144)	0.154 (0.598)	0.415 (0.141)	**0.544** **(0.044)**	0.471 (0.089)	0.412 (0.144)	0.285 (0.323)	0.184 (0.528)	0.255 (0.379)	0.297 (0.303)	0.492 (0.074)	**0.654 (0.040)**	− 0.017 (0.953)	0.079 (0.788)	− 0.287 (0.320)
	DAN → VAN	0.185 (0.526)	0.189 (0.519)	0.393 (0.164)	0.378 (0.182)	0.423 (0.132)	0.310 (0.281)	0.229 (0.430)	0.092 (0.755)	0.172 (0.557)	0.206 (0.481)	0.393 (0.164)	**0.732 (0.016)**	− 0.047 (0.872)	− 0.033 (0.912)	− 0.201 (0.491)
	DAN → FPN	0.396 (0.162)	0.278 (0.337)	0.460 (0.098)	0.367 (0.196)	0.418 (0.137)	0.494 (0.073)	0.301 (0.295)	0.259 (0.370)	0.292 (0.311)	0.354 (0.215)	0.480 (0.082)	0.619 (0.057)	− 0.014 (0.962)	0.138 (0.638)	− 0.306 (0.287)
	DAN → SMN	0.362 (0.204)	0.159 (0.588)	0.367 (0.197)	**0.538** **(0.047)**	0.497 (0.070)	0.334 (0.243)	0.247 (0.394)	0.130 (0.658)	0.238 (0.413)	0.245 (0.399)	0.473 (0.088)	0.622 (0.055)	0.07 (0.812)	0.209 (0.474)	− 0.228 (0.432)

All analyses were conducted with sex, age, and FSIQ as covariates. Bold fonts, nominal significant result

*BRIEF* Behavior Rating Inventory of Executive Function, *WM* working memory, *OM* organization of materials, *TM* task monitor, *EC* emotional control, *MI* metacognition index, *BRI* behavioral regulation index, *DMN* default mode network, *DAN* dorsal attention network, *VAN* ventral attention network, *FPN* frontoparietal network, *SMN* somatomotor network

In the CPT test, the attention performance was positively correlated with the EC values of channels of DAN → VAN and DAN → SMN, respectively. We found that the EC value between C54 → C27 was positively correlated with the 3-digit d′ values (r = 0.585, *P* = 0.028) in the simple + moderate conditions. the EC value between C54 → C49 was positively correlated with the 4-digit d′ values (r = 0.577, *P* = 0.039) in the complex conditions. From the perspective of networks, no significant correlation was found between the score of CPT and EC value changes (Table 2).

### The correlation of EC values with VMI performance

We found that the VMI performance was related to the EC values from DAN to other networks. The EC values between DAN → DMN (r = 0.654, *P* = 0.040) and DAN → VAN (r = 0.732, *P* = 0.016) were positively correlated with the score of Beery VMI in complex conditions. In contrast, no significant correlations were found between the simple + moderate conditions and VMI performance (Table 3). No significant correlation was found between the VMI performance and the EC values of channels.

**Table 3:** The correlation between EC values of channels and lab-based tests in different conditions

Conditions	Networks	BRIEF r (*P*)											VMI scores	CPT r (*P*)		
		WM	Initiate	Plan/organize	OM	TM	Inhibit	Shift	EC	Self-monitor	MI	BRI		2-digit	3-digit	4-digit
Simple + moderate	C48 → C47	− 0.337 (0.26)	− 0.16 (0.602)	− 0.228 (0.454)	− 0.34 (0.256)	− 0.366 (0.219)	− 0.191 (0.532)	− 0.013 (0.965)	− 0.166 (0.587)	− 0.233 (0.444)	− 0.155 (0.613)	− 0.344 (0.25)	0.241 (0.531)	− 0.351 (0.218)	− 0.192 (0.510)	0.219 (0.451)
	C54 → C27	− 0.176 (0.564)	− 0.048 (0.876)	0.009 (0.976)	0.027 (0.930)	− 0.23 (0.45)	− 0.295 (0.328)	− 0.19 (0.534)	− 0.166 (0.588)	− 0.06 (0.846)	− 0.194 (0.526)	− 0.086 (0.779)	− 0.184 (0.635)	0.293 (0.309)	**0.585 (0.028)**	− 0.031 (0.916)
	C54 → C49	− 0.284 (0.347)	0.071 (0.818)	− 0.064 (0.835)	− 0.317 (0.292)	− 0.381 (0.199)	− 0.329 (0.273)	− 0.149 (0.628)	− 0.173 (0.572)	− 0.331 (0.269)	− 0.249 (0.412)	− 0.216 (0.479)	− 0.13 (0.739)	0.343 (0.230)	0.507 (0.064)	0.081 (0.782)
Complex	C48 → C47	− 0.267 (0.356)	0.003 (0.991)	− 0.002 (0.995)	0.089 (0.763)	0.046 (0.875)	− 0.199 (0.496)	− 0.298 (0.301)	− 0.226 (0.437)	0.053 (0.857)	− 0.208 (0.475)	− 0.024 (0.935)	0.023 (0.95)	− 0.041 (0.895)	0.061 (0.844)	− 0.380 (0.200)
	C54 → C27	− 0.009 (0.975)	0.284 (0.326)	0.253 (0.383)	− 0.126 (0.669)	0.285 (0.324)	0.310 (0.281)	0.438 (0.117)	0.198 (0.497)	0.084 (0.775)	0.294 (0.307)	0.177 (0.545)	0.433 (0.211)	− 0.439 (0.133)	− 0.276 (0.361)	0.317 (0.292)
	C54 → C49	− 0.197 (0.500)	0.160 (0.584)	0.180 (0.537)	− 0.021 (0.943)	0.176 (0.546)	0.232 (0.426)	0.200 (0.493)	0.124 (0.673)	0.163 (0.577)	0.192 (0.511)	0.086 (0.771)	0.295 (0.408)	− 0.087 (0.777)	− 0.066 (0.830)	**0.577 (0.039)**

All analyses were conducted with sex, age, and FSIQ as covariates. Bold fonts, nominal significant results

*BRIEF* Behavior Rating Inventory of Executive Function, *WM* working memory, *OM* organization of materials, *TM* task monitor, *EC* emotional control, *MI* metacognition index, *BRI* behavioral regulation index, *CPT* continuous performance test

## Discussion

Our study explored the VMI load-dependent EC values among brain regions during the VMI task and their relationship with VMI performance, attention performance, and EF. Using G-causality analysis, we observed that mean EC values of the DAN → DMN, the DAN → VAN, the DAN → FPN, and the DAN → SMN significantly increased occurring with increasing task difficulty. Specifically, from the perspective of channels, we also found that two brain regions in DAN (right SPL and right middle occipital gyrus), bilateral superior frontal gyrus (SFG) and right postcentral gyrus (PCG) may be related to the VMI activity. Our present findings strongly suggested that the DAN might play an essential role in the processes of visual-motor integration task.

### EC value changes in DAN

According to recent research, the DAN plays a crucial role in top-down spatial attention processing and goal-driven motor planning [45]. Eryurek et al. found that DAN exhibits a high contribution during visuomotor sequence learning tasks, potentially due to the attention demands involved in motor sequence automatization [46]. These findings are consistent with our research results. We found that as the VMI load increased, the EC values between DAN and the DMN, VAN, FPN, and SMN increased. This provides further evidence of the potential involvement of DAN in VMI activities.

The SPL are considered key regions of the DAN and integrates information from visual and somatosensory cortical areas for the execution of reaching and grasping movements [47]. In addition, abnormal activation of SPL has been linked to difficulties with VMI skills. Studies have shown that compared with healthy controls, patients with ASD have abnormal activation of the bilateral SPL and supplementary motor area during visuomotor tasks; the activation abnormalities of these brain regions are thought to be related to less precise visuomotor behavior in ASD [48]. The MOG may also plays a crucial role in the VMI system by integrating visual information for motion control and facilitating the allocation of attentional resources [49, 50]. Sripada et al. found that poor VMI performance showed significant positive relationships with thinner cortical surface area in medial occipital lobe in the very low birth weight young adult patients (19.7 ± 0.9 years old) [51]. In our results, it was found that as the VMI load increased, there is also an increase in the EC between C48 (located in the SPL), C54 (located in the MOG), and other network channels. It suggested that the DAN coordinates with other brain regions to facilitate the selection and processing of relevant visual information, which is crucial for efficient cognitive and motor functioning.

### Effective connectivity between DAN and VAN

The VAN is involved in detecting and processing task-relevant stimuli, and stimulus-driven attentional control [52, 53]. The results of our study indicated that as the task difficulty increased, the EC from the DAN to the VAN was significantly increased. It can be inferred that when the task difficulty increased, the demands for attention and VMI also increased, resulting in increased EC values from the DAN to the VAN. Therefore, the two attention control networks integrate more strongly to support this task more efficiently. These results also provide converging evidence for the theory that the DAN and VAN are not isolated in the process of controlling attention but interact to achieve dynamic control of attention [45, 54].

Specifically, in our study, the C48 → C47 (right SPL → right SFG) and the C54 → C27 (right MOG → left SFG) were all located in the DAN → VAN. From the perspective of channels, we found that the EC values from the right SPL to right SFG, and from the right MOG to left SFG were significantly increased during the more challenging tasks. Previous research has found that the right SPL [13, 55], bilateral SFG [56], and right MOG [12] were all found to be associated with VMI.

The involvement of the SFG in self-awareness, planning, execution of motor control, and attention control has been reported in previous studies [57]. In addition, the SFG has been found to be associated with sensorimotor regions, suggesting its role in the integration of sensory and motor information [58, 59]. Zheng et al. founded that adaptive visuomotor task can enhance EC between the prefrontal and sensorimotor areas [14]. The SPL and SFG have also been implicated in attention control and VMI. Thus, it is reasonable to speculate that when task difficulty increases, the demands for attention and sensorimotor integration also increase, resulting in an increase in EC from the SPL to the SFG. Similarly, Barton et al. found that the increased functional connectivity is related to the difficulty of the writing task and the increase in motor attention demands [18]. These findings are consistent with our results, providing further evidence for the involvement of the SPL and SFG in attention and VMI.

According to partial correlation analysis, we found that in the simple + moderate conditions, the MI of BRIEF and the 3-digit d’ values were positively correlated with EC from DAN → VAN, and in the complex conditions, the VMI behavioral performance was positively correlated with EC from DAN → VAN. Therefore, we speculate that the increased information flow between attention control networks was associated with stronger cognitive control and task monitoring ability during task performance. Attention control may be involved in cognitive control processes at the beginning of the VMI task. As the difficulty increases, attention control may mainly participate in the task monitoring process [60]. In addition, the association between Beery VMI test performance and the EC from DAN to VAN suggested that there was a possible relationship between attention control and VMI performances from the perspective of behavioral performances.

### Effective connectivity between DAN and SMN

According to previous research, the SMN is responsible for controlling voluntary movements, including movements of the arms, hands, and legs [61]. Our findings suggested that the EC values from the DAN to SMN increased during the VMI task. We speculated that more attention is required to perform the VMI task accurately, which leads to an increased effective connection between the DAN and SMN.

PCG, known as the primary somatosensory cortex, integrates visual information about the tool with somatosensory feedback about the body movements. According to a previous study, significant activation of the PCG has been observed in a visuomotor control task, which is believed to be related to VMI [56]. The current findings indicated that EC from the C54 (right MOG, located in the DAN) to the C49 (right PCG, located in the SMN) increased as the task progressed. We hypothesized that EC between the MOG and PCG allows the brain to integrate visual information with other sensory modalities to guide motor behavior. It also adjusts changes in the relationship between visual information and motor behavior caused by increased task difficulty. Furthermore, we also found that in complex conditions, the EC value between DAN → SMN was positively correlated with the Organization of Materials of BRIEF and 4-digit d′ values of CPT. Therefore, we speculated that as the VMI load increased, attention control may mainly participate in the task organization process.

It is believed that the increased activation of the DAN facilitates communication with the SMN, which is responsible for executing the motor commands necessary for the VMI task. Consequently, the EC between the two networks was increased, resulting in improved VMI performance.

### Effective connectivity between DAN and FPN, DMN

Based on our study, we found that as the VMI load increased, the EC from the DAN to the FPN and DMN increased. The FPN is a functional hub that influences brain-wide communication to meet task demands and is involved in executive control. It has extensive connectivity with both the DMN and attention control networks (DAN, VAN), supporting the potential to flexibly couple with either network, depending on task demands [62, 63]. According to our findings, we speculated that the external stimuli lead to an increased need for cognitive functions, resulting in increased information flow between DAN → FPN. This increased information flow then promotes coordinating the completion of VMI activities.

Moreover, DAN is considered a “task-positive” network [64], which is supported by externally-directed attention [65]. In contrast, the DMN is considered a “task-negative” brain network, which is active during self-focused thinking and when it is free from external stimuli. When attention is increasingly focused on external stimuli (e.g., a task becomes more difficult or requires greater cognitive effort), DMN activation decreases [66, 67]. However, our results found that when the task became more difficult, the information flow from the DAN to DMN was increased, which is inconsistent with previous research [68]. One possible explanation is that the increased demand for attention may lead to enhanced top-down control from the DAN to the DMN, which in turn to modulate DMN activity. Specifically, the DAN may suppress the activity of the DMN, allowing for greater cognitive control and more efficient processing of task-relevant information [53]. Further research is needed to fully understand the relationship between the DAN and DMN during different levels of task difficulty. Hence, it can be speculated that the three networks are thought to work together to support VMI by coordinating visual perception and motor action to complete more difficult VMI tasks successfully.

Taken together with the abovementioned evidence of the EC value changes in the VMI task, our findings suggested that attention control plays an important role in VMI activity. Previous studies have found that ADHD patients have significant attention problems and VMI difficulties. Therefore, it may be reasonable to expect that the VMI deficits in ADHD may be caused by attention problems. We will further explore this issue in subsequent studies.

## Limitation

Some limitations should be considered for our present study. Firstly, this study only explored the EC value changes of VMI task in healthy adults, which may not be comprehensive enough to explore the EC features related to VMI-related brain networks. Future research can be applied to neurodevelopmental disorders (such as ADHD and ASD) to discover the characteristics of brain network connectivity associated with VMI activity. Secondly, this was an exploratory study in nature, the correlation analysis between EF, attention performance, and EC values were not corrected for multiple testing. Third, our research did not specifically focus on the impact of scalp blood flow signals and the impact of stress or concentration-related movements (e.g., teeth clenching, facial movements, tension, etc.). Some studies found that compared with non-short channel correction, short channel correction might potentially improve the signal quality and reduce spurious correlations in connectivity measures [69, 70]. In future research, short-channel correction and monitoring other physiological signals (such as heart rate, and electrodermal activity) should be considered to test the same protocol to effectively minimize signal interference and further improve the analyses; Fourth, our study tasks involve eyeball and body movements, such as hand, arm, and head movements. Future research may be able to combine eye tracking and optical sensor technology to simultaneously track the movement information of eyeballs, hands, and heads to conduct more in-depth research on VMI tasks. Fifth, we noticed that the bandpass filtering methods may tend to introduce autocorrelation between time series and lead to missed causalities [71]. To verify our results, we performed the preprocessing without bandpass filtering and spline correction again. We found that the main result hasn't changed, the mean EC values of the DAN → DMN, the DAN → FPN, and the DAN → SMN significantly increased occurring with increasing task difficulty. This finding suggested the robustness of our current results. Future research should use a more comprehensive method to repeat the experiment. Last but not least, the number of participants in our study was relatively small, and studies with larger sample sizes could obtain more convincing results. Therefore, future research needs to repeat the experiment and increase the number of subjects to verify the results.

## Conclusions

In general, our preliminary study showed that DAN may be played an important role in VMI activity. The two key brain regions (right SPL, right MOG) are actively involved the EC value changes of VMI task. Our findings provide a new perspective on the potential precise intervention methods for VMI difficulties in the clinical population.

### Supplementary Information

**Additional file 1.** This file shows the details of Methods and significant EC values of different networks (or channels) among the three conditions.

## Data Availability

The datasets generated and analyzed during the current study are not publicly available due to the subjects’ private information were collected, but are available from the corresponding author on reasonable request.
