# Supplementary material for: Changes in effective connectivity during the visual-motor integration tasks: a preliminary f-NIRS study
Source: Behav Brain Funct. 2024 Mar 11;20:4. doi: 10.1186/s12993-024-00232-3 (PMC10929220; doi:10.1186/s12993-024-00232-3)
Supplement: Supplementary file 1 — Additional file 1. This file shows the details of Methods and significant EC values of different networks (or channels) among the three conditions. [file 12993_2024_232_MOESM1_ESM.pdf]

Appendix

Methods

Table S1. The description of demographic characteristics

	Healthy participants (N=23)
**Sex**	
**Male (n, %)**	10 (43.48)
**Female (n, %)**	13 (56.52)
**Age (Mean ± SD)**	24.74 ± 3.00
**FSIQ (Mean ± SD)**	123.57 ± 5.98

NOTE. FSIQ: full-scale intelligence quotient.

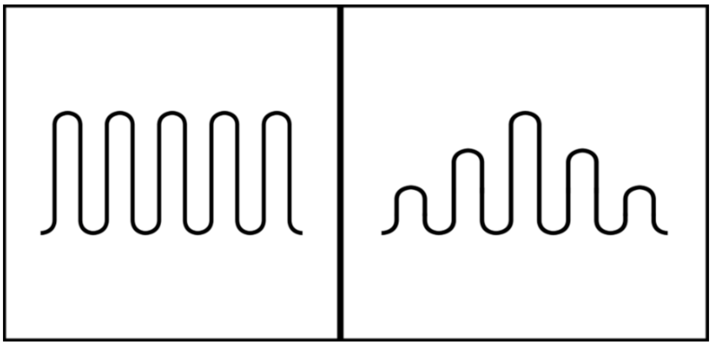

Figure S1. The practice figures.

The method of confirming the positions of each measurement channel

We randomly choose a participant for structural MRI scanning. We labeled all the source-detector positions of the fNIRS cap using the vitamin E capsules, the Vitamin E were used as landmarks for coregistration. Then, their T1-weighted structural image was acquired using a General Electric; Discovery MR750 3.0 Tesla scanner. The MR image was normalized into Montreal Neurological Institute (MNI) space using the NIRS_SPM software [1]. And then, the MNI coordinates were determined according to the automated anatomical labeling (AAL) template [2].

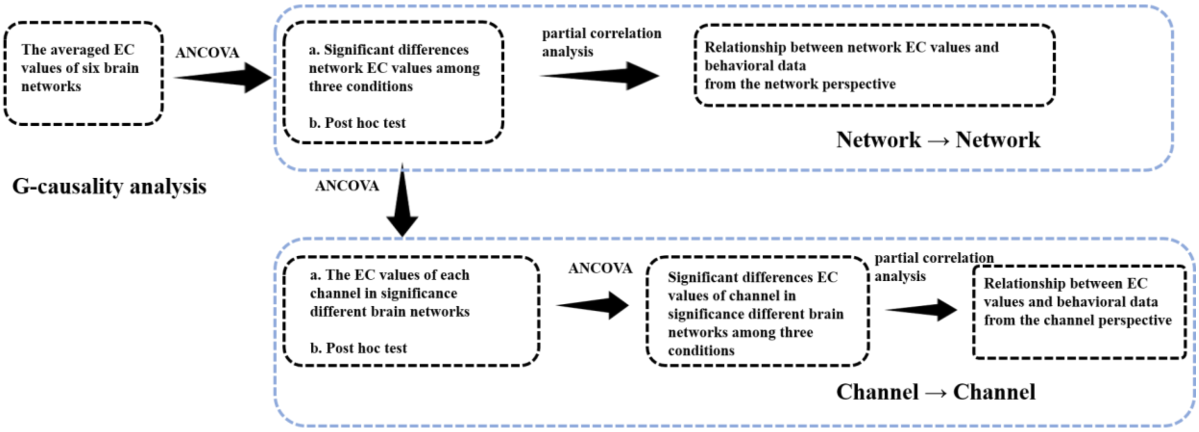

Figure S2. The flow chart for the GC analyses

Table S2. The MNI coordinates and the distribution of all channels in the six brain networks.

channel	x	y	z	Brain networks	Hemisphere
1	-39.00	58.67	16.00	Frontoparietal	L
2	-52.33	40.67	11.33	Frontoparietal	L
3	-49.00	40.67	24.67	Frontoparietal	L
4	-34.67	35.67	46.67	Frontoparietal	L
5	-23.67	54.67	37.67	Default_Mode	L
6	-32.33	56.67	27.33	Frontoparietal	L
7	-42.67	38.67	36.67	Frontoparietal	L
8	-66.00	-9.67	33.33	Somatomotor	L
9	-60.67	16.00	18.00	Default_Mode	L
10	-58.00	14.67	28.67	Default_Mode	L
11	-25.33	32.33	55.67	Default_Mode	L
12	-18.33	29.67	61.33	Default_Mode	L
13	-14.67	51.67	45.67	Default_Mode	L
14	-42.67	14.33	57.33	Default_Mode	L
15	-57.00	-9.00	50.00	Somatomotor	L
16	-49.33	-12.33	58.67	Somatomotor	L
17	-49.00	17.00	46.67	Default_Mode	L
18	-35.67	12.67	63.00	Default_Mode	L
19	-27.33	10.33	67.33	Frontoparietal	L
20	-42.00	-14.00	67.00	Somatomotor	L
21	-33.67	-16.00	73.00	Somatomotor	L
22	-64.00	-34.67	46.33	Ventral_Attention	L
23	-65.00	-45.33	37.33	Frontoparietal	L
24	-54.67	-35.67	56.33	Dorsal_Attention	L
25	-59.67	-56.67	40.33	Default_Mode	L
26	-53.00	-59.33	51.33	Default_Mode	L
27	-17.33	3.33	73.00	Ventral_Attention	L
28	-23.67	-18.67	76.00	Somatomotor	L
29	-45.33	-34.67	66.67	Somatomotor	L
30	-42.67	-58.67	59.67	Frontoparietal	L
31	-32.67	-57.67	69.00	Dorsal_Attention	L
32	-35.33	-37.67	72.00	Somatomotor	L
33	-51.67	-77.33	27.67	Default_Mode	L
34	-44.33	-79.33	39.33	Default_Mode	L
35	-33.67	-93.67	22.33	Visual	L
36	-22.00	-57.33	73.67	Dorsal_Attention	L
37	-22.67	-39.33	76.00	Somatomotor	L
38	-36.67	-79.67	47.67	Default_Mode	L
39	-25.33	-77.33	55.33	Dorsal_Attention	L
40	-25.33	-92.67	32.33	Visual	L

41	22.67	-54.67	74.00	Dorsal_Attention	R
42	26.00	-35.00	76.00	Somatomotor	R
43	24.33	-76.33	58.67	Dorsal_Attention	R
44	35.67	-78.33	48.67	Frontoparietal	R
45	22.33	-93.67	32.67	Visual	R
46	25.67	-17.33	75.33	Somatomotor	R
47	19.33	6.00	72.67	Ventral_Attention	R
48	33.33	-56.33	70.00	Dorsal_Attention	R
49	36.33	-35.67	72.00	Somatomotor	R
50	44.33	-57.67	59.67	Frontoparietal	R
51	47.33	-33.33	65.67	Somatomotor	R
52	44.67	-78.00	39.67	Frontoparietal	R
53	31.67	-94.33	22.33	Visual	R
54	51.33	-76.67	27.67	Dorsal_Attention	R
55	36.00	-14.00	71.67	Somatomotor	R
56	30.33	12.33	65.33	Frontoparietal	R
57	45.67	-10.67	64.33	Somatomotor	R
58	40.67	15.67	59.67	Frontoparietal	R
59	54.33	-58.33	51.33	Frontoparietal	R
60	58.33	-33.67	56.33	Frontoparietal	R
61	66.00	-43.33	39.33	Frontoparietal	R
62	61.00	-56.33	40.33	Default_Mode	R
63	66.33	-32.33	46.67	Ventral_Attention	R
64	21.67	32.67	59.00	Default_Mode	R
65	19.33	51.67	45.33	Default_Mode	R
66	30.67	35.67	52.00	Default_Mode	R
67	54.33	-8.67	55.67	Somatomotor	R
68	52.33	19.33	42.67	Frontoparietal	R
69	47.67	17.67	52.33	Frontoparietal	R
70	61.33	-6.33	45.67	Somatomotor	R
71	27.33	53.33	38.33	Frontoparietal	R
72	34.67	57.67	26.33	Frontoparietal	R
73	44.33	40.33	36.33	Frontoparietal	R
74	38.33	37.33	45.33	Frontoparietal	R
75	63.00	16.67	17.33	Frontoparietal	R
76	59.67	17.67	28.33	Frontoparietal	R
77	68.00	-6.67	32.33	Somatomotor	R
78	42.67	58.67	13.67	Frontoparietal	R
79	56.00	39.33	10.67	Frontoparietal	R
80	52.33	40.67	23.33	Frontoparietal	R

RESULTS

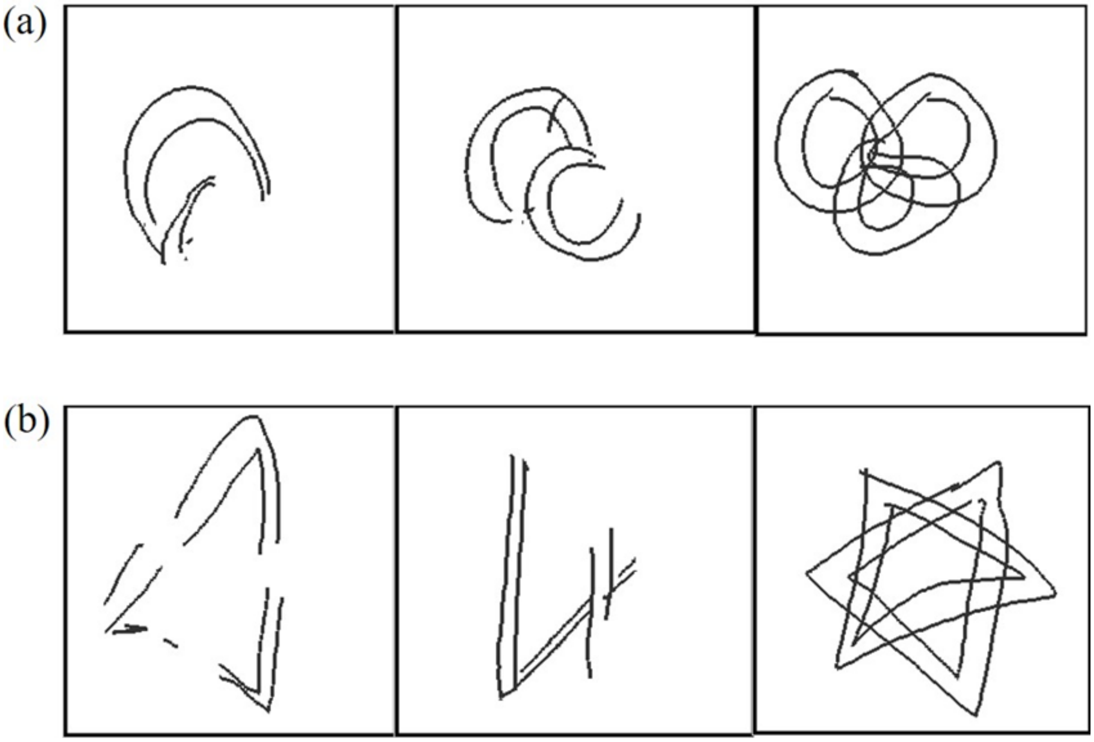

Figure S3. The results of the inaccurate drawing pictures. (a) the incomplete drawings of Picture 21 for three subjects; (b) the incomplete drawings of Picture 24 for three subjects.

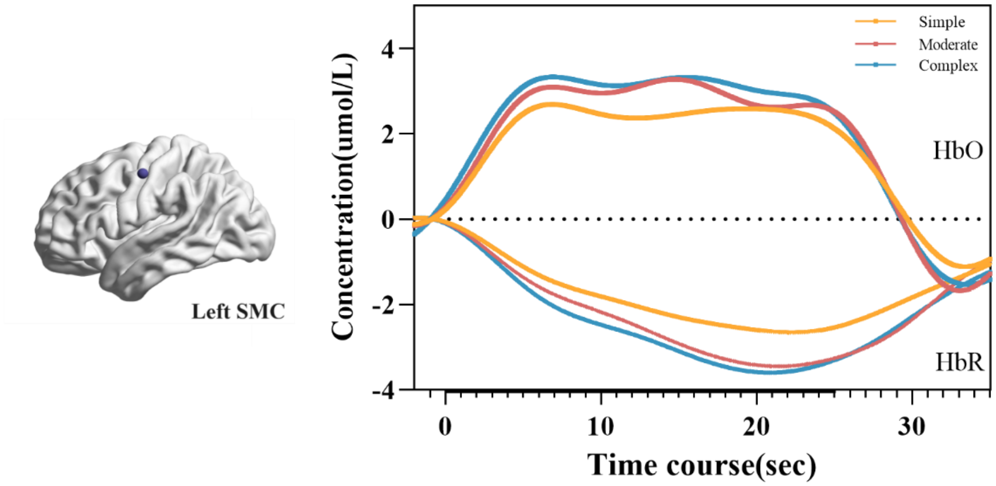

FigureS4. The time course of the fNIRS response (both HbO and HbR)

Mean HbO and HbR concentration evolving from 2 s pre-stimulus to 35 s post-stimulus for the three task conditions in channel #15 (located in the left SMC) are shown as an example. The horizontal bold black line denotes the period of task execution. SMC: sensorimotor cortex.

TableS3. Significantly different EC values of brain networks among the three conditions

Network→Network	EC values			F	*P*	Post hoc
	Simple	Moderate	Complex			(Bonferroni)
DAN→DMN	0.082±0.062	0.082±0.055	0.159±0.084	7.160	0.002	1,2<3
DAN→VAN	0.088±0.086	0.083±0.058	0.169±0.083	6.701	0.003	1,2<3
DAN→FPN	0.095±0.072	0.082±0.055	0.159±0.075	6.093	0.004	1,2<3
DAN→SMN	0.078±0.063	0.102±0.069	0.172±0.097	6.703	0.003	1,2<3

Note. EC: effective connectivity. DMN: default mode network; DAN: dorsal attention network; VAN: ventral attention network; FPN: frontoparietal network; SMN: somatomotor network

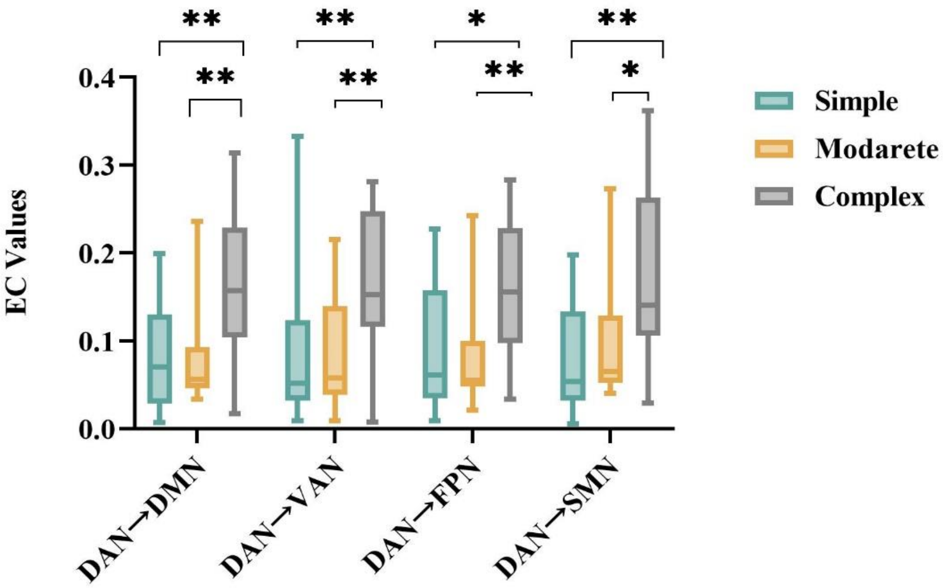

Fig. S5. Significantly EC values of different networks among the three conditions (post-hoc analysis). Note. EC: effective connectivity. DMN: default mode network; DAN: dorsal attention network; VAN: ventral attention network; FPN: frontoparietal network; SMN: somatomotor network. * $P < 0.05$, ** $P < 0.01$

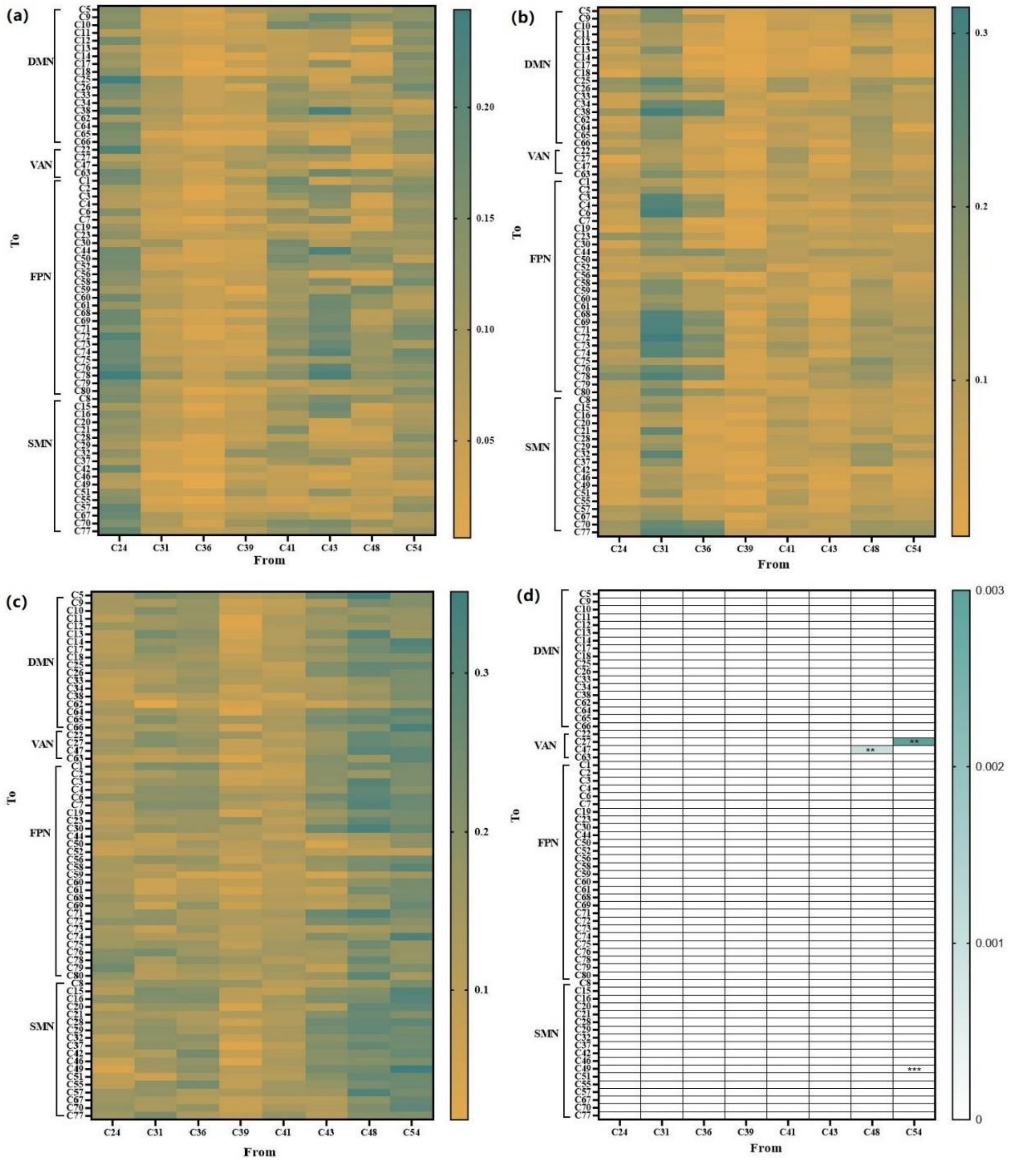

Figure S6. EC matrix for all channels in the brain network with significant differences. (a) simple condition; (b) moderate condition; (c) complex condition; * represents significant differences survived FDR correction for multiple testing (**$P < 0.01$; *** $P < 0.001$). **Note.** DMN: default mode network; DAN: dorsal attention network; VAN: ventral attention network; FPN: frontoparietal network; SMN: somatomotor network.

Table S4. Significantly different EC values of channels among the three conditions

Network	Channel	EC values			F	*P*	Post hoc (Bonferroni)	AAL
		simple	moderate	complex				
DAN→VAN	C48→C47	0.023±0.035	0.088±0.159	0.294±0.328	7.566	0.001	1,2<3	Parietal_Sup_R→Frontal_Sup_R
	C54→C27	0.045±0.063	0.049±0.083	0.248±0.307	6.527	0.003	1,2<3	Occipital_Mid_R→Frontal_Sup_L
DAN→SMN	C54→C49	0.044±0.091	0.046±0.093	0.351±0.320	13.322	2.499E-05	1,2<3	Occipital_Mid_R→Postcentral_R

Note. EC: effective connectivity. DAN: dorsal attention network; VAN: ventral attention network; SMN: somatomotor network. Parietal_Sup_R: right superior parietal lobule; Frontal_Sup_R: right superior frontal gyrus; Occipital_Mid_R, right middle occipital gyrus; Frontal_Sup_L, right superior frontal gyrus; Postcentral_R: right postcentral gyrus.

REFERENCE:

1. Ye JC, Tak S, Jang KE, Jung J, & Jang J. NIRS-SPM: statistical parametric mapping for near-infrared spectroscopy. NeuroImage. 2009; 44, 428-47
2. Tzourio-Mazoyer N, Landeau B, Papathanassiou D, Crivello F, Etard O, Delcroix N, et al. Automated anatomical labeling of activations in SPM using a macroscopic anatomical parcellation of the MNI MRI single-subject brain. NeuroImage. 2002; 15, 273-89
